# Combining modified Graeb score and intracerebral hemorrhage score to predict poor outcome in patients with spontaneous intracerebral hemorrhage undergoing surgical treatment

**DOI:** 10.3389/fneur.2022.915370

**Published:** 2022-07-29

**Authors:** Shen Wang, Xuxu Xu, Qiang Yu, Haicheng Hu, Chao Han, Ruhai Wang

**Affiliations:** ^1^Department of Neurosurgery, Shanghai University of Medicine and Health Sciences Affiliated Jia Ding Hospital, Shanghai, China; ^2^Department of Neurosurgery, Shanghai Minhang District Central Hospital, Shanghai, China; ^3^Department of Neurosurgery, Fuyang Fifth People's Hospital, Anhui, China

**Keywords:** modified Graeb score, intracerebral hemorrhage score, surgical treatment, outcome, spontaneous intracerebral hemorrhage

## Abstract

**Objective:**

Spontaneous intracerebral hemorrhage (sICH) is a frequently encountered neurosurgical disease. The purpose of this study was to evaluate the relationship between modified Graeb Score (mGS) at admission and clinical outcomes of sICH and to investigate whether the combination of ICH score could improve the accuracy of outcome prediction.

**Methods:**

We retrospectively reviewed the medical records of 511 patients who underwent surgery for sICH between January 2017 and June 2021. Patient outcome was evaluated by the Glasgow Outcome Scale (GOS) score at 3 months following sICH, where a GOS score of 1–3 was defined as a poor prognosis. Univariate and multivariate logistic regression analyses were conducted to determine risk factors for unfavorable clinical outcomes. Receiver operating characteristic (ROC) curve analysis was performed to detect the optimal cutoff value of mGS for predicting clinical outcomes. An ICH score combining mGS was created, and the performance of the ICH score combining mGS was assessed for discriminative ability.

**Results:**

Multivariate analysis demonstrated that a higher mGS score was an independent predictor for poor prognosis (odds ratio [OR] 1.207, 95% confidence interval [CI], 1.130–1.290, *p* < 0.001). In ROC analysis, an optimal cutoff value of mGS to predict the clinical outcome at 3 months after sICH was 11 (*p* < 0.001). An increasing ICH-mGS score was associated with increased poor functional outcome. Combining ICH score with mGS resulted in an area under the curve (AUC) of 0.790, *p* < 0.001.

**Conclusion:**

mGS was an independent risk factor for poor outcome and it had an additive predictive value for outcome in patients with sICH. Compared with the ICH score and mGS alone, the ICH score combined with mGS revealed a significantly higher discriminative ability for predicting postoperative outcome.

## Introduction

Spontaneous intracerebral hemorrhage (sICH) is defined as non-traumatic bleeding into the brain parenchyma and accounts for 10–30% of all strokes (1, 2), with a high rate of disability and mortality (3). Surgical treatment is one of the main treatments for ICH. At present, multiple surgical techniques are utilized to treat ICH, including craniotomy, minimally invasive surgery, and decompressive craniectomy (4). The ICH score is a clinical grading method that can be used to predict 30-day mortality in patients with ICH. This score, which consists mainly of the Glasgow Coma Scale (GCS) score, patient age, and neuroimaging features, is effective in predicting the short-term risk of death or poor outcome in patients with ICH (5).

It has been reported in the literature that ICH is often complicated by intraventricular hemorrhage (IVH), which can further exacerbate brain damage and thus influence the prognosis (6). The modified Graeb Score (mGS) is used as an indicator to assess the severity of IVH and is calculated based on three factors, namely, location of IVH, hematoma volume in each ventricle, and ventricular dilatation, as described by Morgan et al. (7), and has a high predictive value for the prognosis of IVH.

However, the relationship between mGS and sICH has not been widely recognized, so the aim of this study was to evaluate the relationship between mGS at admission and clinical outcomes of sICH and to investigate whether the combination of ICH score could improve the accuracy of outcome prediction.

## Methods

### Patient population

We retrospectively reviewed the medical records of 511 patients who underwent surgery for sICH between January 2017 and June 2021, in two hospitals (Shanghai University of Medicine and Health Sciences Affiliated Jia Ding Hospital in Shanghai and Fuyang Fifth People's Hospital in Anhui Province, China). The inclusion criteria were as follows: 1. all patients were older than 18 years. 2. patients had ICH with or without IVH on head computed tomography (CT) scan. 3. patients underwent a hematoma evacuation; and 4. patients had complete documentation. The exclusion criteria were as follows: 1. ICH caused by head trauma, brain tumor, cerebral aneurysm, and vascular malformation. 2. missing imaging data. 3. previous history of ICH or other neurological diseases such as ischemic stroke. 4. other systematic diseases such as renal dysfunction, hepatic dysfunction, cancer, hematological disorders, and heart diseases; and 5. patients who were lost to follow-up at 3 months. This study was performed in accordance with the principles of the Declaration of Helsinki, and the protocol was approved by the Ethics Committee of the Fuyang Fifth People's Hospital.

### Data collection

Data collected included gender, age, ICH location, hypertension and/or diabetes, blood pressure at admission, time from symptom onset to baseline CT scan, antiplatelet agents use, GCS score at admission, and preoperative findings such as cerebral hernia, presence of obstructive hydrocephalus, IVH, mGS, and ICH score. An operative method such as external ventricular drainage (EVD) was considered for analysis. The postoperative findings including acute cerebral infarction and intracranial infection were investigated.

The diagnosis of intracranial infection defined according to the standards issued by the National Ministry of Health is as follows: 1) presence of clinical manifestation of intracranial infection, including temperature higher than 38°C or lower than 36°C, positive signs of meningeal irritation (nuchal rigidity, Brudzinski sign, and Kernig sign), vomiting, and headache. 2) positive changes in cerebrospinal fluid specimens: white blood cell count > 1,000 × 10^6^ cells/L; glucose levels < 2.25 mmol/L; chloride < 120 mmol/L, and protein > 0.45 g/L; and 3) positive results for bacteria in cerebrospinal fluid culture (8). Cerebral infarction is defined as radiologically proven new infarcts.

### Imaging

Two experienced neurologists were employed to independently review all the CT scans in a blinded manner. All patients underwent baseline CT within 6 h after onset of symptoms, and follow-up CT scan was performed within 24 h after the baseline CT scan. Hematoma volumes were calculated by the ABC/2 method (9). In this study, we defined hematoma growth as an increase in hematoma volume of >33% or >6 ml at follow-up CT scan (10, 11). In addition, IVH growth was defined as either any newly occurring intraventricular bleeding on follow-up CT scan in patients without baseline IVH or an increase in IVH volume ≥1 ml on follow-up CT scan in patients with initial IVH (12).

### Outcome assessment

The mGS and ICH scores at admission were calculated to assess the severity of ICH. All included patients with sICH were treated surgically, of which 215 cases received EVD. All patients with sICH were treated stringently following the guidelines for the management of sICH (13). Patient outcome was evaluated by the Glasgow Outcome Scale (GOS) score at 3 months following sICH. Good outcomes were defined as GOS score of 4–5, and score of 1–3 was deemed a poor outcome (14).

### Statistical analysis

Statistical analysis was performed using SPSS version 26.0 (SPSS, Inc., Chicago, USA). Categorical variables were presented as numbers with percentages and analyzed with an χ^2^ test or Fisher's exact test, whereas continuous variables were expressed as mean ± standard deviation and analyzed using Student's *t*-test or Mann–Whitney *U* test. Univariate and multivariate logistic regression analyses were conducted to determine the risk factors for unfavorable clinical outcomes. Variance inflation factors (VIFs) were used to examine independent variables for potentially strong contributions to multicollinearity in a regression model, and it was suggested that predictors with values above a VIF > 5 or a tolerance index < 0.20 could be contributing considerably to multicollinearity (15). Receiver operating characteristic (ROC) curve analysis was performed to detect the optimal cutoff value of mGS for predicting clinical outcomes. To assess the prognostic capability of ICH score combining mGS (ICH-mGS score), the area under the curve (AUC) was calculated. Z-test was used to compare the difference of AUC between the ICH score and ICH-mGS score. ROC analysis was also used to estimate the predictive accuracy of the ICH-mGS score on the IVH growth of patients with sICH. Statistical significance was defined as *p* < 0.05 for all tests.

## Results

A total of 511 patients were included in this study. There were 268 men and 243 women, with an average age of 62.86 ± 9.66 years. ICH location was 465 (91.0%) supratentorial and 46 (9.0%) infratentorial. The admission GCS score was 7.86 ± 2.92. Notably, 102 (20.0%) patients had cerebral hernia, 84 (16.4%) patients had obstructive hydrocephalus, and 300 (58.7%) patients had associated IVH. Of note, 157 (30.7%) patients with ICH experienced hematoma expansion (HE), and 124 (24.3%) patients had IVH growth on follow-up CT scan. The admission mGS score was 7.91 ± 8.18 and the ICH score was 1.88 ± 0.92. Notably, 215 (42.1%) patients underwent EVD placement. The clinical characteristics, in detail, of the 511 patients are listed in Table 1.

**Table 1:** Clinical characteristics of 511 patients with sICH.

**Characteristics**	**Value**
**Number of patients**	511
Gender, *n* (%)	
Male	268 (52.4)
Female	243 (47.6)
Age, mean ± SD (years)	62.86 ± 9.66
Medical history	
Hypertension, *n* (%)	292 (57.1)
Diabetes mellitus, *n* (%)	38 (7.4)
Antiplatelet agents use, *n* (%)	41 (8.0)
ICH location, *n* (%)	
supratentorial	465 (91.0)
infratentorial	46 (9.0)
Systolic blood pressure (mmHg), mean ± SD	165.50 ± 9.35
Diastolic blood pressure (mmHg), mean ± SD	97.51 ± 10.33
Time from onset to CT (h), mean ± SD	1.26 ± 0.54
Preoperative finding	
GCS score at admission, mean ± SD	7.86 ± 2.92
Cerebral hernia, *n* (%)	102 (20.0)
Obstructive hydrocephalus, *n* (%)	84 (16.4)
Intraventricular hemorrhage, *n* (%)	300 (58.7)
mGS, mean ± SD	7.91 ± 8.18
ICH score, mean ± SD	1.88 ± 0.92
Hematoma growth, *n* (%)	157 (30.7)
IVH growth, *n* (%)	124 (24.3)
EVD, *n* (%)	215 (42.1)
Postoperative finding	
Acute cerebral infarction, *n* (%)	8 (1.6)
Intracranial infection, *n* (%)	17 (3.3)
GOS after 3 months, *n* (%)	
1–3	183 (35.8)
4–5	328 (64.2)

*sICH, spontaneous intracerebral hemorrhage; SD, standard deviation; CT, computed tomography; GCS, Glasgow coma scale; mGS, modified Graeb Score; IVH, intraventricular hemorrhage; EVD, external ventricular drainage; GOS, Glasgow outcome scale*.

At 3 months after sICH, an unfavorable clinical outcome was found in 183 (35.8%) patients and a favorable prognosis was found in 328 (64.2%) patients. The poor outcome group showed lower initial GCS and higher rate of women than the good outcome group. The poor outcome group had higher rates of those with cerebral hernia, obstructive hydrocephalus, IVH, hematoma growth, IVH growth, EVD, and intracranial infection than the good outcome group. The poor outcome group had higher age, mGS, and ICH score than the good outcome group. Between groups, there were no significant differences in ICH location, medical history, antiplatelet agents use, admission blood pressure, time from symptom onset to baseline CT scan, and postoperative cerebral infarction (Table 2).

**Table 2:** Baseline characteristics of patients with good and poor outcome group.

**Characteristics**	**Good outcome**	**Poor outcome**	***p*** **-value**
Number of patients	328 (64.2)	183 (35.8)	
Gender, *n* (%)			0.003^*^
Male	188 (57.3)	80 (43.7)	
Female	140 (42.7)	103 (56.3)	
Age, mean ± SD (years)	61.30 ± 8.89	65.67 ± 10.36	<0.001^*^
Medical history			
Hypertension, *n* (%)	187 (57.0)	105 (57.4)	0.936
Diabetes mellitus, *n* (%)	29 (8.8)	9 (4.9)	0.105
Antiplatelet agents use, *n* (%)	30 (9.1)	11 (6.0)	0.211
ICH location, *n* (%)			0.415
supratentorial	301 (91.8)	164 (89.6)	
infratentorial	27 (8.2)	19 (10.4)	
Systolic blood pressure (mmHg), mean ± SD	164.89 ± 9.15	166.58 ± 9.62	0.061
Diastolic blood pressure (mmHg), mean ± SD	96.82 ± 10.04	98.74 ± 10.75	0.059
Time from onset to CT (h), mean ± SD	1.24 ± 0.54	1.31 ± 0.55	0.357
Preoperative finding			
GCS score at admission, mean ± SD	8.78 ± 2.76	6.21 ± 2.42	<0.001^*^
Cerebral hernia, *n* (%)	34 (10.4)	68 (37.2)	<0.001^*^
Obstructive hydrocephalus, *n* (%)	29 (8.8)	55 (30.1)	<0.001^*^
Intraventricular hemorrhage, *n* (%)	162 (49.4)	138 (75.4)	<0.001^*^
mGS, mean ± SD	5.46 ± 6.51	12.29 ± 9.01	<0.001^*^
ICH score, mean ± SD	1.57 ± 0.79	2.44 ± 0.87	<0.001^*^
Hematoma growth, *n* (%)	70 (21.3)	87 (47.5)	<0.001^*^
IVH growth, *n* (%)	58 (17.7)	66 (36.1)	<0.001^*^
EVD, *n* (%)	113 (34.5)	102 (55.7)	<0.001^*^
Postoperative finding			
Acute cerebral infarction, *n* (%)	2 (0.6)	6 (3.3)	0.050
Intracranial infection, *n* (%)	5 (1.5)	12 (6.6)	0.002^*^

SD, standard deviation; ICH, intracerebral hemorrhage; CT, computed tomography; GCS, Glasgow coma scale; mGS, modified Graeb Score; IVH, intraventricular hemorrhage; EVD, external ventricular drainage.

* p < 0.05 was considered significant.

Multicollinearity was not observed between the independent variables studied and outcome. A multivariate logistic regression analysis revealed that gender [odds ratio (OR), 2.315; 95% confidence interval (CI), 1.378–3.889; *p* = 0.002], age (OR, 1.074; 95% CI, 1.041–1.108; *p* < 0.001), GCS score at admission (OR, 0.790; 95% CI, 0.698–0.893; *p* < 0.001), IVH (OR, 0.191; 95% CI, 0.069–0.528; *p* = 0.001), mGS (OR, 1.207; 95% CI, 1.130–1.290; *p* < 0.001), ICH score (OR, 1.961; 95% CI, 1.159–3.317; *p* = 0.012), hematoma growth (OR, 3.395; 95% CI, 1.624–7.101; *p* = 0.001), EVD (OR, 0.482; 95% CI, 0.238–0.977; *p* = 0.043), and intracranial infection (OR, 6.866; 95% CI, 1.142–41.266; *p* = 0.035) were independent risk factors for poor outcome of sICH (Table 3).

**Table 3:** Multivariate logistic regression analysis of predictors for poor outcome.

**Variable**	**OR**	**95% CI**	**VIF**	***p-*** **value**
Gender Age	2.315 1.074	1.378–3.889 1.041–1.108	1.124 1.149	0.002 <0.001
GCS score at admission Intraventricular hemorrhage mGS ICH score Hematoma growth EVD Intracranial infection	0.790 0.191 1.207 1.961 3.395 0.482 6.866	0.698–0.893 0.069–0.528 1.130–1.290 1.159–3.317 1.624–7.101 0.238–0.977 1.142–41.266	1.927 3.729 3.911 2.837 1.065 2.042 1.069	<0.001 0.001 <0.001 0.012 0.001 0.043 0.035

*OR, odds ratio; CI, confidence interval; VIF, variance inflation factor; GCS, Glasgow coma scale; mGS, modified Graeb Score; ICH, intracerebral hemorrhage; EVD, external ventricular drainage*.

**Figure 1:**
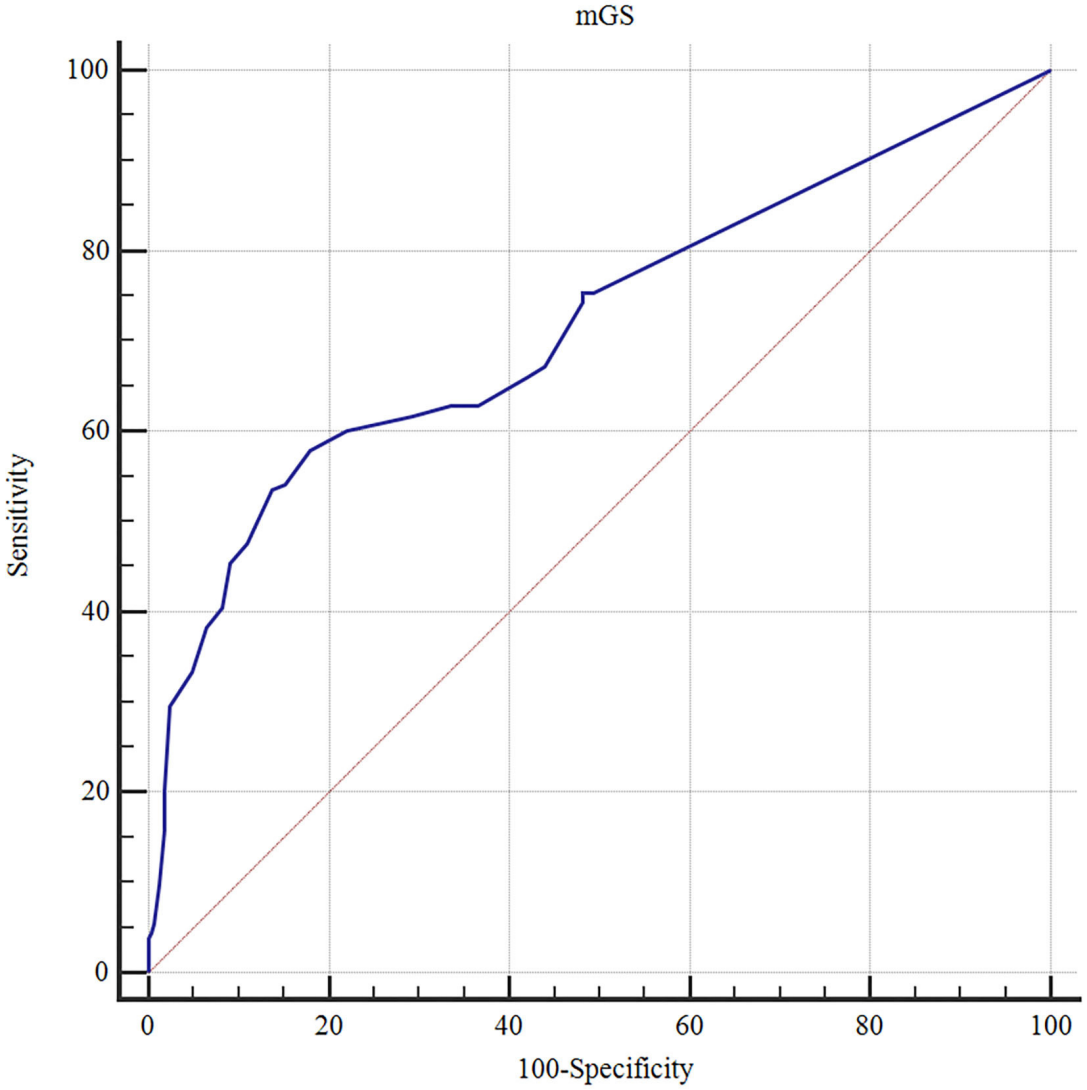
Receiver operating characteristic (ROC) curve of mGS score on the poor outcome of sICH.

**Figure 2:**
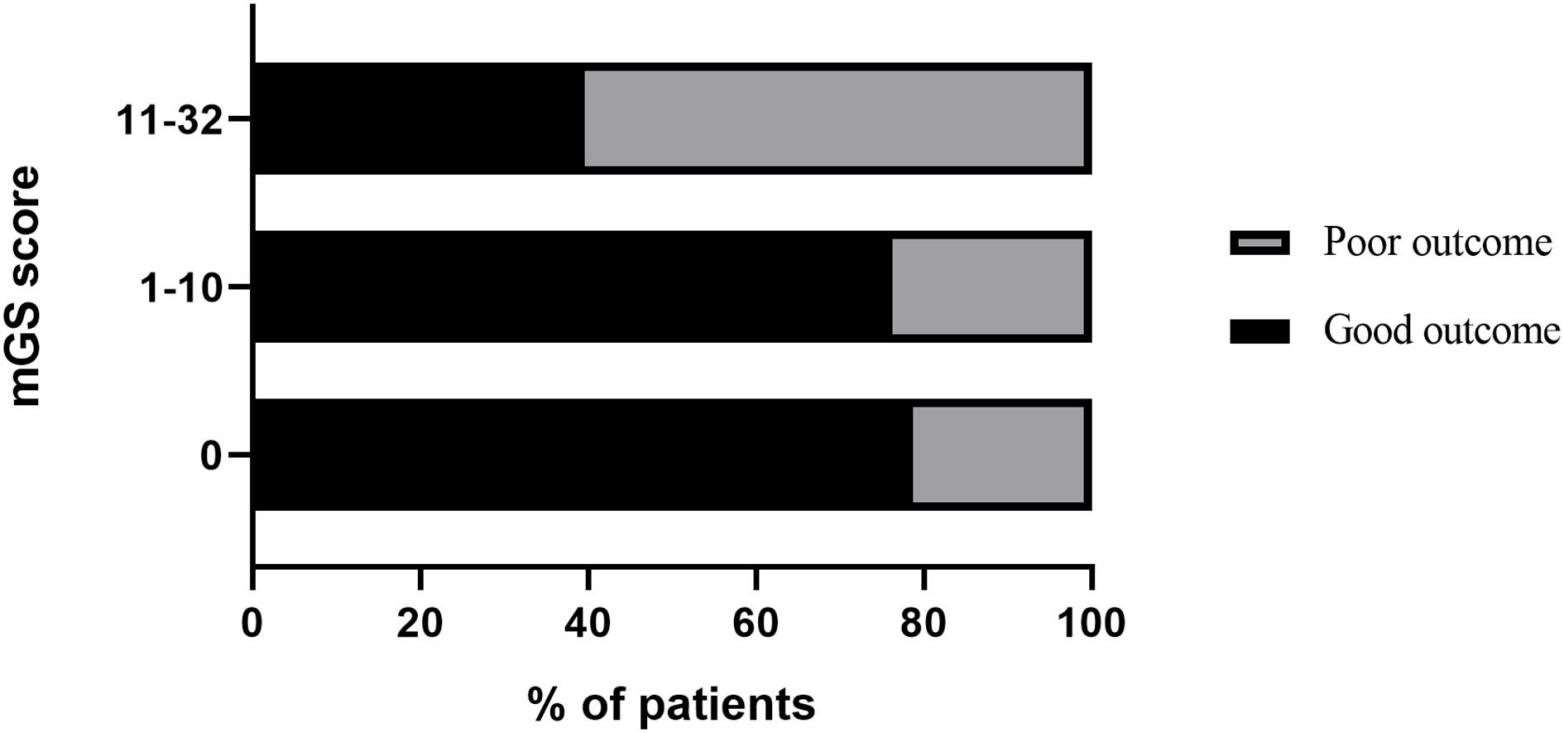
Probability of poor functional outcome at 3 months for mGS.

The ROC curves analysis showed that the optimal cutoff value of mGS to predict the clinical outcome at 3 months after sICH was 11 (AUC: 0.718; 95% CI, 0.677–0.756; *p* < 0.001) (Figure 1). The probability of poor functional outcome at 3 months for mGS score is shown in Figure 2. We found that in the different mGS score groups, the higher the mGS score, the higher the proportion of patients with poor outcome.

**Figure 3:**
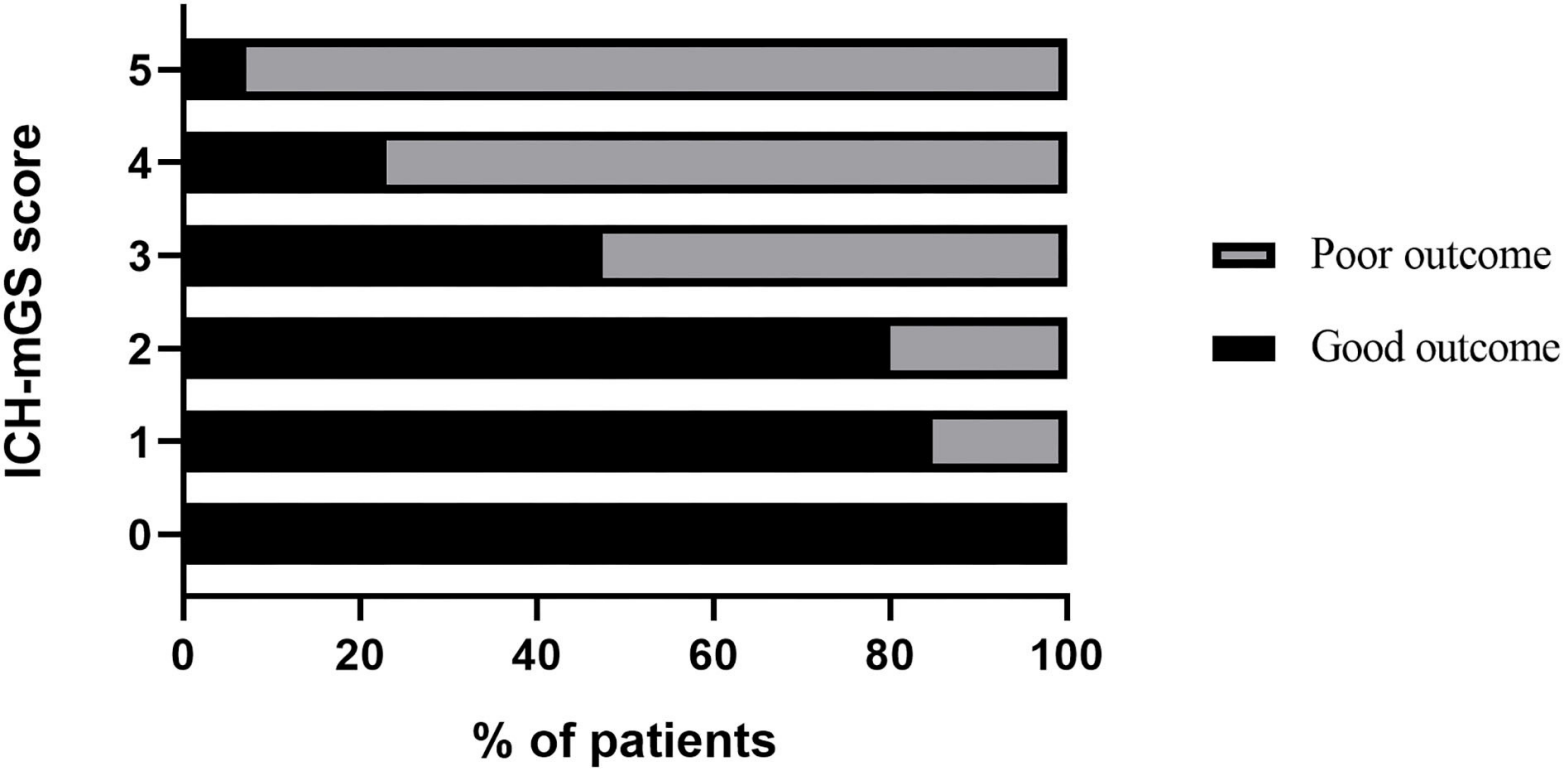
Probability of poor functional outcome at 3 months for ICH-mGS score.

**Figure 4:**
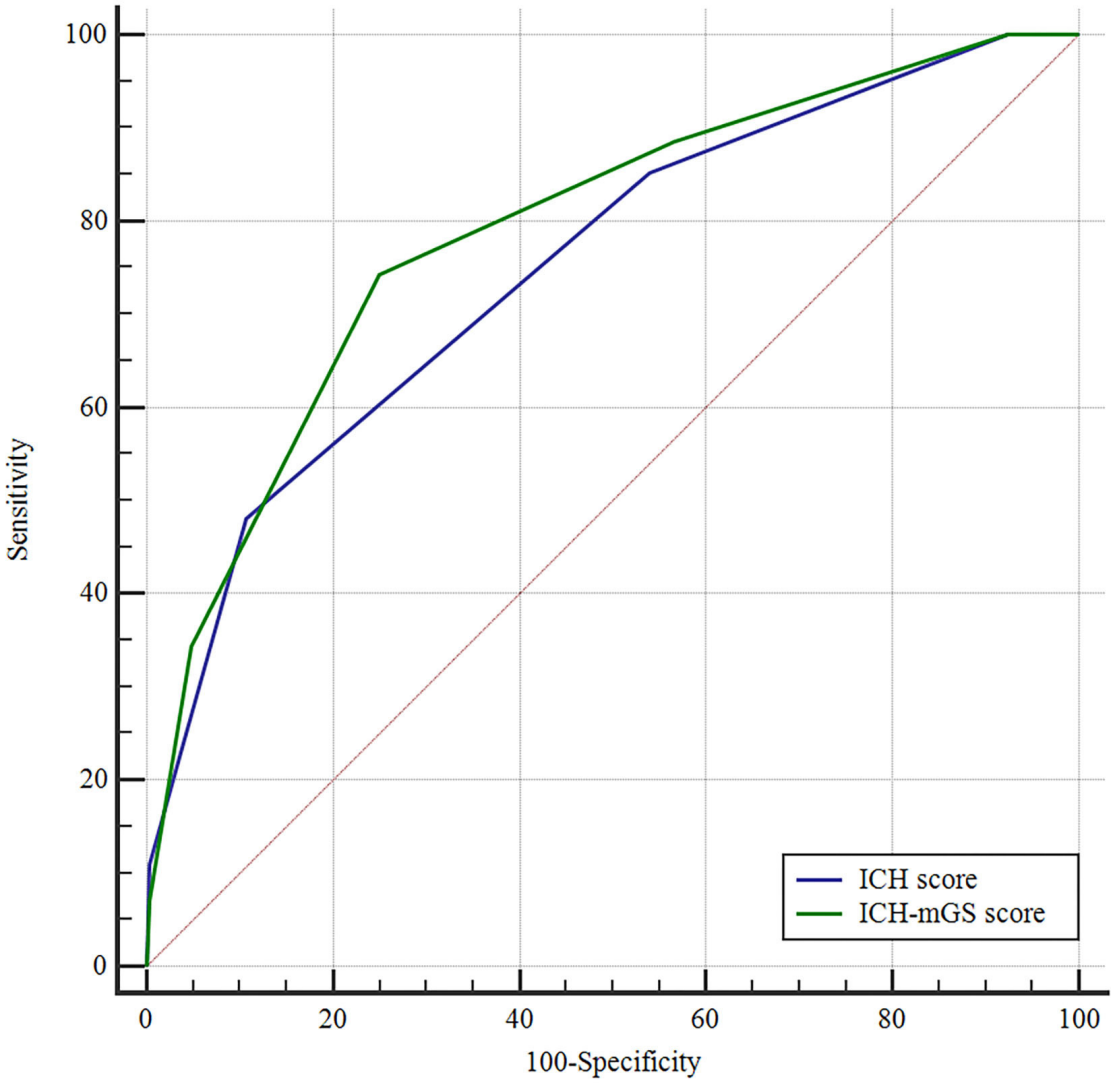
Receiver operating characteristic (ROC) curves of ICH score, ICH-mGS score on the poor outcome of sICH.

The mGS scores were divided into 3 subgroups (0, 1–10, and 11–32) and assigned 0, 1, or 2 points, respectively, to assess their additive predictive ability to the ICH score. We replaced IVH in the ICH score with mGS and designed an ICH score combining mGS with a total score ranging from 0 to 7 points (Table 4). The probability of poor functional outcome at 3 months for the ICH-mGS score is shown in Figure 3. Poor outcomes were observed, respectively, in 0.00%, 15.22%, 20.00%, 52.52%, 76.92%, and 92.86% of the patients. ROC curves were created to assess the ability of ICH-mGS score to predict poorer functional outcome. In ROC analysis, the AUC of ICH-mGS score predicted poor outcome of sICH was 0.790 (cutoff value = 2; 95% CI, 0.752–0.824; *p* < 0.001), and AUC of ICH score was 0.751 (cutoff value = 2; 95% CI, 0.712–0.788; *p* < 0.001) (Figure 4). Compared with the ICH score alone, the ICH-mGS score was significantly better for predicting an unfavorable outcome (Z = 3.795, *p* < 0.001).

**Table 4:** ICH score combining mGS.

**Item**	**ICH–mGS score (points)**
GCS score	
3–4 5–12 13–15	2 1 0
ICH volume (mL)	
≥ 30 <30	1 0
Infratentorial origin of ICH	
Yes No	1 0
Age (years)	
≥ 80 <80	1 0
mGS	
0 1–10 11–32	0 1 2
Total score	0–7

*ICH, intracerebral hemorrhage; mGS, modified Graeb Score; GCS, Glasgow coma scale*.

**Figure 5:**
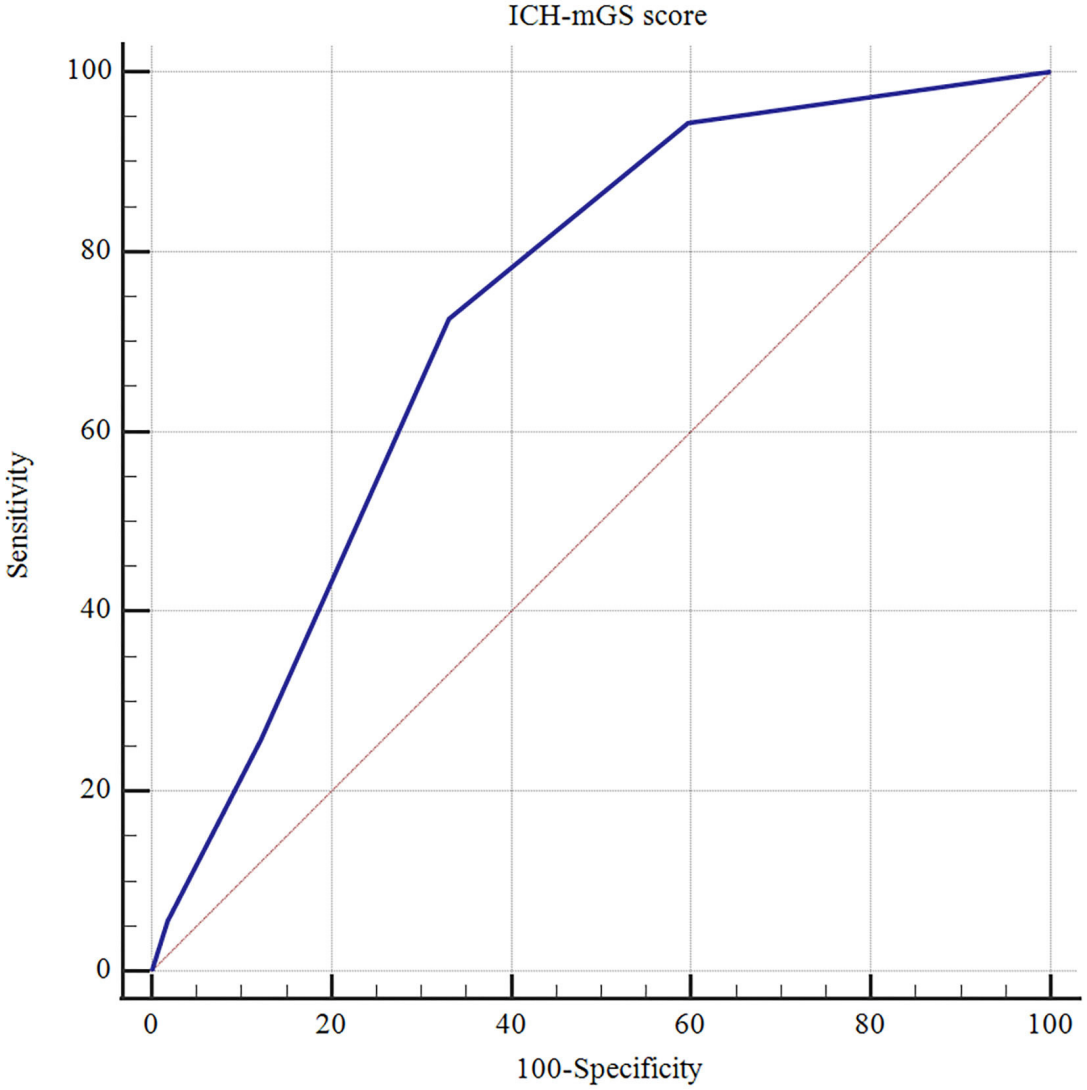
Receiver operating characteristic (ROC) curve of ICH-mGS score on the IVH growth.

An ROC curve analysis was also made to estimate the predictive ability of the ICH-mGS score for IVH growth. An ICH-mGS score of 2 was observed to have the best cutoff value with AUC of 0.734 (95% CI, 0.693–0.772; *p* < 0.001), sensitivity of 72.58%, and specificity of 66.93% (Figure 5).

## Discussion

This study demonstrated that a higher mGS score at admission was an independent predictor of the prognosis of sICH, mGS had significant prognostic value for unfavorable outcomes following a surgical treatment of sICH, and combining mGS and ICH score was of greater value in predicting poor prognosis.

Intracerebral hemorrhage is a common critical disease in neurosurgery. Surgical intervention is one of the main treatments for ICH. Traditionally, open craniotomy and hematoma aspiration were considered the mainstay of surgical management of ICH. In recent years, some of the surgical approaches that have been studied include stereotactic guidance aspiration and thrombolysis, image-guided stereotactic endoscopic aspiration, ultrasound-induced thrombolysis, stereotactic intracerebral hemorrhage underwater blood aspiration technique, and minimally invasive surgery (16). Increasing evidence suggests that newer surgical techniques have positive therapeutic effects on ICH and can be more effective than the conventional treatments (17, 18). The annual incidence rate of ICH is found as high as 35 persons per 100,000 individuals per year (19), and its high disability and mortality rates impose a huge burden on families and society (20). An identification of risk factors associated with poor outcome of ICH is of paramount importance for the treatment. Factors such as age, initial GCS, ICH volume, and diabetes mellitus are commonly associated with ICH (21–24). IVH is one of the common complications of ICH and is an independent predictor of worse outcome and neurological deterioration (25–27); the reason for this is that prolonged exposure of the ventricles to blood may lead to altered consciousness and, at the tissue level, inflammation, fibrosis, and hydrocephalus (28, 29).

Thurim et al. (30) first demonstrated that IVH volume was related to higher 30-day mortality rates. Young et al. (27) identified that IVH volume of 20 ml was a predictive value for poor outcome. In addition, Hwang et al. (31) reported that admission IVH volume of 6 ml was associated with a significant increase in the likelihood of poor functional outcome after sICH. Thus, this suggested that the amount of IVH was a key factor in the prognosis of ICH. The mGS score is used as a semiquantitative scale for IVH volume measurement with a total score of 0–32 and is similarly predictive of outcome to actual IVH volume (7). There is another method available for the assessment of IVH volume, and the Graeb scale is highly accurate to prognosticate the risk of mortality and long-term disability in patients with ICH (6). In contrast, compared to the original Graeb Scale (oGS), mGS is more reliable in predicting poor prognosis, following ICH. Hansen et al. (32) found that the mGS improved outcome prediction after supratentorial ICH beyond other established prognostic factors in a broad ICH population. Morgan et al. (7) reported that mGS was more closely related to change in IVH volume and outcome than the oGS. In this study, the mGS score was significantly higher in the GOS poor outcome group than in the good outcome group, the optimal cutoff value of mGS was 11 to predict the clinical outcome (AUC: 0.718, 95% CI: 0.677–0.756, *p* < 0.001), and from univariate and multivariate regression analyses, mGS was a risk factor for poor prognosis. Therefore, the mGS score serves as an assessment tool for IVH severity and can be readily used to predict the poor clinical outcomes of patients with sICH after surgical treatment.

The ICH score is a prognostic tool commonly used in clinical practice that includes GCS score, age ≥ 80 years, infratentorial location, hematoma volume, and presence of IVH, and is used to evaluate 30-day mortality of patients with ICH. The ICH score ranges from 0 to 6, with 30-day mortality rates increasing from 0% for a score of 0 to 100% for score of 5 or 6 (5). This study first combines mGS and ICH score and sets a new ICH-mGS score for evaluating poor outcome in patients with sICH. In our study, both mGS and ICH score were found to be independent predictors for poor outcome in sICH, an increasing ICH-mGS score was associated with increased poor functional outcome, and in ROC analysis, the AUC for the combined mGS and ICH score was better than that of each parameter alone, suggesting that the ICH-mGS score showed a potential advantage to predict poorer functional outcome relative to ICH score and mGS.

Hematoma expansion (HE) is a common early and severe complication of ICH (33, 34). Several studies indicate that HE occurs in approximately one-third of patients with ICH and is associated with in-hospital mortality and poor outcome (35–37). At present, the relevant imaging markers have been used to predict HE after ICH, including spot sign, leakage sign, island sign, and intra-hematomal hypodensity on non-contrast CT (38). IVH growth is most often defined as either the development of delayed IVH or an increase in IVH volume, individually. Previous studies have reported that IVH growth is an independent predictor of death and poor functional outcome in patients with ICH (12, 39, 40). Li et al. (12) reported that IVH growth occurred at 19.5% and was an independent predictor of poor functional outcome at 3 months after ICH. Dowlatshahi et al. (41) demonstrated that patients (6.1%) without HE had ≥2 ml IVH expansion, which was associated with poor outcome. In addition, other studies have shown that IVH growth is strongly associated with early HE (6). Patients who had severe HE could develop delayed IVH subsequently, which ultimately led to poor clinical outcomes (42). We argue that IVH growth might be a complication of HE, and IVH growth together with hematoma growth can result in a worse outcome in patients with ICH. In this study, we found that HE was a risk factor for poor prognosis; however, IVH growth was not independently associated with an unfavorable outcome following ICH. ROC curve was further applied, and the results showed that the ICH-mGS score could also significantly predict the IVH growth. Thus, our findings suggest that the ICH-mGS score reveals a higher discriminative ability for predicting the IVH growth and poor outcome in patients with sICH undergoing surgical treatment.

Consistent with the previous reports, we found that the admission GCS score was associated with poor clinical outcome. Øie et al. (43) reported that a GCS score of < 9 on admission was a risk factor for poor prognosis at 3 months in patients with ICH. Wang et al. (44) also found that patients with a GCS score of 3 to 4 had a significantly higher 30-day mortality than those with a GCS score of 5 to 15. In our study, multivariate analysis showed that a low GCS score was associated with poor outcome of patients with sICH after hematoma evacuation. In addition, we found that gender, age, IVH, EVD, and intracranial infection were related to poor outcome of sICH.

Neuroinflammation has been linked to neurological diseases, especially cerebrovascular disease (45). Previous studies have revealed that inflammatory response plays a pivotal role in the pathologic mechanism of ICH and is associated with poor functional outcome (46, 47). A number of biochemical factors, including white blood cell count, C-reactive protein, tumor necrosis factor alpha, vascular endothelial growth factor, homocysteine, and neutrophil-to-lymphocyte ratio (NLR), are known as inflammatory markers in ICH (48–52). There is increasing evidence that easily available serum biomarkers of inflammation can be reliable predictors of outcome in patients with ICH and can improve the outcome prediction when added to validated prognostic scales (53, 54). Lattanzi et al. (53) reported that NLR was associated with 30-day mortality and morbidity after ICH, and improved the accuracy of outcome prediction when added to the modified ICH score. Zhou et al. (55) demonstrated that elevated plasma D-dimer level was an independent risk factor for poor functional outcome, and the ICH outcome score combining D-dimer level could effectively evaluate and predict mortality at 3 months after ICH. Taken together, we propose that a combination of clinical grading scales and serum inflammatory biomarkers could aid to more exhaustively explain and refine the ICH prognosis and should be further analyzed for its potential to predict the outcome after ICH.

Our study has several limitations. First, this was a retrospective study, the selection bias may exist, and given the subselection of only surgical patients, which may affect the catholicity of the study results. Second, this study did not assess the role of hematological parameters in the prognosis of sICH. Third, the number of patients available for analysis was small, and no patients showed the ICH-mGS score of 6–7, making the assessment of outcome less reliable. Further studies with a prospective and multicenter trial are required to confirm these findings.

## Conclusion

Our study showed that mGS was an independent risk factor for poor outcome and it had an additive predictive value for outcome in patients with sICH undergoing surgical treatment. Compared with the ICH score and mGS alone, the ICH score combined with mGS exhibited a better predictive ability for the functional prognosis of patients with sICH undergoing surgical treatment.

## Data availability statement

The original contributions presented in the study are included in the article/supplementary material, further inquiries can be directed to the corresponding author.

## Ethics statement

The studies involving human participants were reviewed and approved by the Ethics Committee of the Fuyang Fifth People's Hospital. The patients/participants provided their written informed consent to participate in this study.

## Author contributions

Conception or design of the study: SW and RW. Data collection: SW, QY, and RW. Data analysis and interpretation: SW, XX, and CH. Drafting this article: SW. Critical revision of this article: RW. Others (study supervision, funding, materials): HH and RW. All authors reviewed the results and approved the final version of the manuscript.

## Conflict of interest

The authors declare that the research was conducted in the absence of any commercial or financial relationships that could be construed as a potential conflict of interest.

## Publisher's note

All claims expressed in this article are solely those of the authors and do not necessarily represent those of their affiliated organizations, or those of the publisher, the editors and the reviewers. Any product that may be evaluated in this article, or claim that may be made by its manufacturer, is not guaranteed or endorsed by the publisher.

## Abbreviations

sICH: spontaneous intracerebral hemorrhage
GCS: Glasgow coma scale
IVH: intraventricular hemorrhage
mGS: modified Graeb Score
CT: computed tomography
EVD: external ventricular drainage
GOS: Glasgow outcome scale
VIF: variance inflation factor
ICH-mGS score: ICH score combining mGS
ROC: receiver operating characteristic
AUC: area under the curve
OR: odds ratio
CI: confidence interval
oGS: original Graeb Scale
HE: hematoma expansion
NLR: neutrophil-to-lymphocyte ratio.
